# Neutrophil-lymphocyte Ratio Plus Prognostic Nutritional Index Predicts the Outcomes of Patients with Unresectable Hepatocellular Carcinoma After Transarterial Chemoembolization

**DOI:** 10.1038/s41598-017-13239-w

**Published:** 2017-10-24

**Authors:** Chang Liu, Lei Li, Wu-sheng Lu, Hua Du, Lu-nan Yan, Jia-yin Yang, Tian-fu Wen, Guo-jun Zeng, Li Jiang, Jian Yang

**Affiliations:** 1 Department of Liver Surgery and Liver Transplantation Centre, West China Hospital, Sichuan University, Chengdu, 610041 China; 2 Department of Vascular Surgery, West China Hospital, Sichuan University, Chengdu, 610041 China

## Abstract

For many malignancies, inflammation-based scores correlate with survival. The neutrophil-to-lymphocyte ratio (NLR) and prognostic nutritional index (PNI) are immunonutritional indices associated with postoperative outcomes in patients with hepatocellular carcinoma (HCC). We evaluated whether a combined preoperative NLR and PNI score was prognostically superior to either index alone in 793 patients with unresectable HCC after transarterial chemoembolization. Patient demographic, clinical, and pathological data were also collected and analysed. A receiver-operating characteristic (ROC) analysis was used to classify patients as follows: NLR-PNI 0 group (NLR ≤ 2.2 and PNI > 46), NLR-PNI 1 group (NLR > 2.2 or PNI ≤ 46) and NLR-PNI 2 group (NLR > 2.2 and PNI ≤ 46). Regarding 1-, 3-, and 5-year survival, the NLR-PNI score had superior discriminative abilities (i.e., higher area under the ROC curve), compared with either the NLR or PNI alone, and patients in the NLR-PNI 0, 1, and 2 groups had median survival times of 33 (95% confidence interval: 22.8–43.2), 14 (10.9–17.1), and 6 (9.9–14.1) months, respectively. In multivariate analyses, the Barcelona Clinic Liver Cancer, total bilirubin, vascular invasion, and NLR-PNI score adversely affected overall survival. In conclusion, the NLR-PNI score can improve the accuracy of prognoses for patients with unresectable HCC.

## Introduction

Hepatocellular carcinoma (HCC) is the most common primary liver cancer and the third leading cause of cancer-related mortality worldwide^1^. Additionally, HCC was estimated to be the second leading cause of cancer-related death among men^2^. Currently, the prevalence of hepatitis B virus (HBV) infection in China is high, and more than 50% of HCC cases and deaths have been estimated to occur in this country^2^. However, a majority (60%) of patients with HCC have an advanced disease stage or liver decompensation at diagnosis and are therefore ineligible for curative therapies such as hepatic resection, liver transplantation, and frequency ablation. Rather, locoregional therapies such as transarterial chemoembolization (TACE) are widely used to treat unresectable intermediate and advanced HCCs^3^.

TACE exploits the progressive tumour angiogenesis and thus targets neoplastic tissue intra-arterially via the focused administration of chemotherapy administration, followed by the induction of acute ischemic damage with relative sparing of the surrounding tissue^4^. Evidence from randomized controlled trials has demonstrated the efficacy of TACE for improving overall survival (OS) by slowing disease progression^5^. However, studies have also shown that not all patients with unresectable HCC benefit from TACE. Therefore, it is crucial to differentiate between those patients who will and will not likely benefit from TACE.

The presence of an inflammatory response is considered pathogenic in the development of cancer-associated malnutrition, and leads to a poor performance status and increased mortality in cancer patients. Particularly, this is a concern for patients with HCC in China because the majority of HCC cases are consequent to an underlying chronic HBV infection, and the concomitant underlying illness and cirrhosis may contribute to an impaired nutritional status^6^. Researchers have expressed increasing interest in the role of systemic inflammation as a predictor of the outcomes of patients with HCC. Apart from objective markers of systemic inflammation, such as the C-reactive protein (CRP) and albumin (ALB) levels, various combinations of haematological components of the systemic inflammatory response have widely investigated, including the neutrophil to lymphocyte ratio (NLR) and prognostic nutritional index (PNI). However, the predictive abilities of the NLR and PNI regarding the post-TACE outcomes of patients with HCC remain unclear. Huang *et al*.^7^ revealed that an elevated NLR was associated with poor overall survival (OS) among patients undergoing TACE, and Pinato *et al*.^6^, who first proposed the PNI, proved that a lower score is an independent and externally validated predictor of poor OS in patients with HCC. However, Kinoshita *et al*.^8^ suggested that neither the NLR nor the PNI correlated with the outcomes of HCC. In addition, He *et al*.^9^ confirmed that the NLR and PNI are less powerful predictive systems that for patients receiving TACE for unresectable HCC.

To date, few studies have evaluated the relationship between the NLR and PNI to predict the outcomes of intermediate to advanced HCC, especially in a prognostic model based on a combination of these indices. Therefore, it remains unclear whether a combination of inflammation-based scores could better reflect the systemic inflammatory state. The present study investigated whether a combination of the NLR and PNI would be a useful prognostic predictor of the postoperative outcomes of TACE-treated patients with unresectable HCC.

## Patients and Methods

### Patient characteristics

Patients with newly diagnosed intermediate-to-advanced HCC who received TACE as an initial therapy at the Department of Liver Surgery, West China Hospital, Sichuan University (Chengdu, China) between January 2007 and December 2013 were enrolled in this study. The diagnosis of HCC was based on the diagnostic criteria used by the American Association for the Study of the Liver^10^. Patients who fulfilled all of the following criteria were included: (1) no treatment before TACE; (2) Child–Pugh liver function of A or B; (3) follow-up period ≥1 month; and (4) HCC was deemed unresectable by a liver cancer multidisciplinary team comprising surgeons, oncologists, radiologists, and interventional radiologists. Patients were excluded if they underwent hepatic resection, radio-frequency treatment, liver transplantation, or another appropriate treatment for HCC during the follow-up period. This study was approved by the Institutional Review Board (IRB) of the West China Hospital, Sichuan University. Informed written consent was obtained from all individual participants included in the study. All the methods used in this study were carried out according to the approved guidelines.

Clinicopathologic variables, including demographic parameters; full blood counts; ALB, total bilirubin (TB), alpha-fetoprotein (AFP), alanine aminotransferase (ALT), and aspartate aminotransferase (AST) levels; prothrombin time (PT); international normalized ratio (INR); tumour staging information (including number of focal lesions and maximum diameter of contrast-enhancing lesions); Child–Turcotte–Pugh class; Model for End-stage Liver Disease (MELD) score; and Barcelona Clinic Liver Cancer (BCLC) stage^11^, were collected at the time of referral to our department, prior to treatment.

### Inflammation-based index and score

The NLR was defined as the absolute neutrophil count divided by the lymphocyte count^12^. The PNI was calculated as follows: albumin (g/L) + 0.005 × absolute lymphocyte count (mm^3^)^13^. The NLR and PNI cut-off values were determined using receiver operating characteristic (ROC) curves according to the patients’ OS status.

A new inflammation-based score system, the NLR-PNI score, was then generated by combining the NLR score with the PNI score. The NLR-PNI score was calculated from pre-TACE data as follows: patients with both an elevated NLR and a decreased PNI were allocated a score of 2, those with either an elevated NLR or a decreased PNI were allocated a score of 1, and those with a decreased NLR and an elevated PNI were allocated a score of 0.

### TACE Procedures

A uniform treatment protocol was applied to each patient. TACE was performed through the femoral artery via the Seldinger technique with local anaesthesia. After arteriography of the celiac trunk and superior mesenteric artery to visualize arterial vascularization of the liver, body surface-dependent doses of the chemotherapeutic agents 5-fluorouracil (800–1000 mg) and epirubicin-adriamycin (30–40 mg) were injected. Subsequently, lipiodol (Lipiodol Ultra-Fluide; Andre Guerbet Laboratories, France) and polyvinyl alcohol foam (PVA) embolization particles (Cook, Bloomington, IN, USA, 100–500 μm in diameter) were injected as selectively as possible into the hepatic segmental artery at the target tumour location. The embolization agent doses ranged from 5 to 30 mL and were determined based on the tumour location, size, and number.

### Follow up

One month after TACE, all patients underwent blood cell tests, liver function tests, AFP measurements, computed tomography or magnetic resonance imaging, and chest radiography to evaluate tumour responses. If elevated tumour marker levels (AFP), diminished lipiodol, enlarged lesions, or new nodules were observed, the patients were readmitted for angiography and treatment within an interval of 1.5–3.0 months. If a patient either could not tolerate the procedure because of a decline in his/her clinical status or presented with a complete response, TACE was terminated. The starting point of the survival analysis was defined as the day of the initial treatment. The end of follow-up was recorded as either December 31, 2013 (the last follow-up) or the date of death. Patients who died within 30 days of the procedure were defined as periprocedural mortalities and were excluded from the post-TACE survival analysis.

### Statistics

Two-group comparisons were conducted using Student’s t test for continuous data. The chi-square test and Fisher’s exact test were used to compare categorical variables. The NLR and PNI cut-off values predictive of overall survival, as well as the discriminatory ability of each scoring system at 1, 3, and 5 years of follow-up, were calculated using ROC curves. OS was analysed using the Kaplan–Meier method, and significant inter-group differences were identified using the log-rank test. The prognostic variables predictive of OS were assessed using a multivariate Cox proportional hazards regression analysis. Variables that were significant in the univariate analysis were subsequently tested in the multivariate Cox proportional hazard model using a forward selection method. All statistical tests were two sided, and a significant difference was defined as a p value < 0.05. All statistical analyses were performed using the IBM SPSS Statistics software package, version 24.0 (IBM SPSS Inc., Chicago, IL, USA).

## Results

### Cut-off values for the inflammation based index

**Figure 1:**
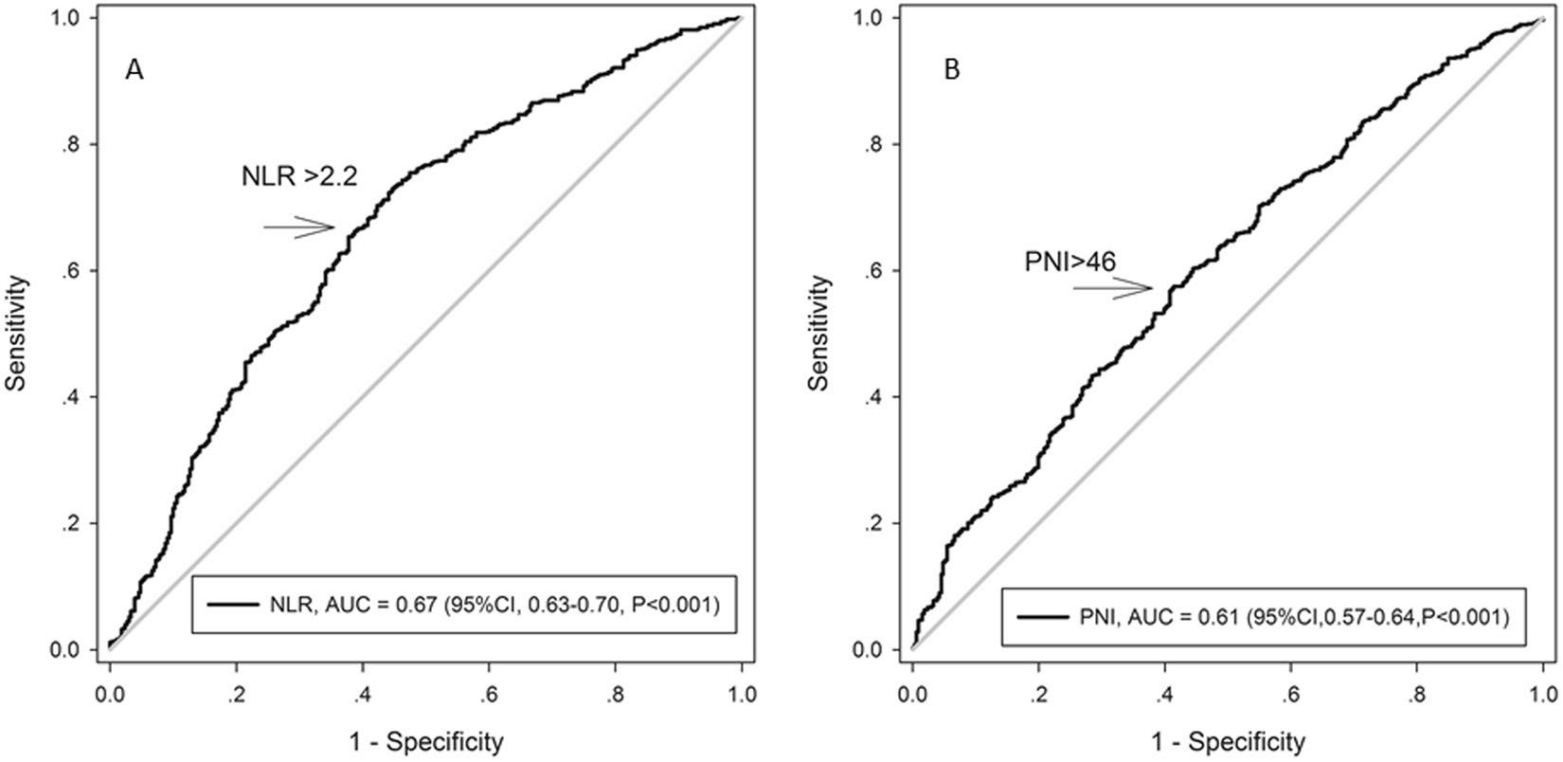
The optimal cut-off value of NLR and PNI on the Receiver operating characteristic curve to predict the overall survive. A: NLR; B: PNI.

A time-dependent ROC analysis was used to determine NLR and PNI cut-off values of 2.2 and 46, respectively (Fig. 1), and these values were used to calculate the NLR-PNI score for each patients. Subsequently, patients were divided into the NLR-PNI 0, NLR-PNI 1, and NLR-PNI 2 groups, as described above.

### Baseline characteristics

This study included 793 patients with unresectable HCC who had undergone TACE during the study period and for whom complete data were available. The sample included 665 men (83.9%) and 128 women (16.1%) with a median age of 56 years (range, 19–89 years). The median values and ranges of the pre-TACE white blood cell, neutrophil, lymphocyte, and platelet counts, as well as the NLR and PNI scores, are shown in Table 1. Most patients (82.1%) were classified as having preserved liver function (Child–Pugh class A). The most common aetiology of cirrhosis was hepatitis B infection (84.9%). Overall, 54.2% of the patients received >1 TACE treatment (range: 1–9). Among the 793 included patients, 139 (17.6%), 308 (38.8%), and 346 (43.6%) patients were assigned into the NLR-PNI 0, NLR-PNI 1, and NLR-PNI 2 groups, respectively. Patients in the NLR-PNI 2 group had a relatively higher AST level, PT value, and largest tumour size, compared with the other groups (P < 0.001). The three groups had significantly different ALB levels, and patients in the NLR-PNI 0 group had the highest level (P < 0.001). The demographic and clinicopathologic features of the three groups are listed in Table 2.

**Table 1:** Values for total white blood cells, neutrophils, lymphocytes, platelet counts, NLR and PNI.

Blood components	Mean	Median	Mininum	Maximum	Normal value
Total white blood cells (×10^9^/L)	5.8 ± 2.7	5.3	2.1	19.4	4.00–10.00
Absolute neutrophil count (×10^9^/L)	3.9 ± 2.4	3.5	0.7	12.7	1.80–6.40
Absolute lymphocyte count (×10^9^/L)	1.1 ± 0.6	1.2	0.4	4.2	1.00–3.30
Total platelets (×10^9^/L)	139.8 ± 88.9	122.5	56	935	100–300
NLR	3.9 ± 3.4	2.9	0.7	10.25	
PNI	44.6 ± 7.6	44.7	29.9	71.1	

WBC = white blood cells; NLR = neutrophil-to-lymphocyte ratio; PNI = prognosis nutritional index.

**Table 2:** Comparison of the clinical characteristics of patients with different NLR-PNI score.

Variable	NLR-PNI 0 (n = 139)	NLR-PNI 1 (n = 308)	NLR-PNI 2 (n = 346)	P value
Age (y)	55.5 ± 13.3	54.0 ± 13.9	56.2 ± 13.4	0.121
Gender (male/female)	118/21	263/45	284/62	0.603
BMI (Kg/m^2)^	22.1 ± 3.1	22.6 ± 3.3	22.3 ± 2.9	0.109
HBsAg (positive/negative)	125/14	254/54	294/52	0.125
ALT (IU/L)	59.2 ± 50.8	60.5 ± 63.7	57.3 ± 49.9	0.761
AST (IU/L)	59.1 ± 42.9	71.4 ± 63.8	89.2 ± 74.6	<0.001^a^
TBIL (μmol/L)	18.2 ± 13.6	19.4 ± 13.3	25.3 ± 17.5	0.005
ALB (g/L)	43.3 ± 6.2	41.1 ± 8.6	35.3 ± 4.4	<0.001^b^
PT (s)	12.0 ± 1.4	12.4 ± 1.8	13.0 ± 1.6	<0.001^a^
INR	1.1 ± 0.1	1.1 ± 0.1	1.1 ± 0.1	0.856
Creatinine (μmol/L)	78.2 ± 15.7	79.6 ± 64.3	73.9 ± 20.7	0.233
Child-pugh grade (A/B)	135/4	272/36	244/102	<0.001b
MELD score	6.0 ± 6.3	5.4 ± 3.9	6.3 ± 3.9	0.076
AFP (μg/L), ≤400/>400	87/52	182/126	181/165	0.067
Largest tumor size (cm)	6.3 ± 3.5	7.5 ± 3.8	9.0 ± 4.7	<0.001a
Tumor number (single/multiple)	55/84	131/177	122/214	0.83
Vascular invasion (absent/present)	90/49	155/153	138/208	0.002^b^
TACE treatments (1/2/>2)	55/34/50	133/89/86	175/85/86	0.058

^a^P < 0.05, when NLR-PNI 2 *vs*. NLR-PNI 0 and NLR-PNI 1; ^b^P < 0.05 when each group compared with each other. NLR = neutrophil-to-lymphocyte ratio, WBC = white blood cells, PLT = platelets, ALT = alanine transaminase, AST = aspartate transaminase, HBsAg = hepatitis B surface antigen, AFP = α-fetoprotein, TBIl = total bilirubin, ALB = albumin, PT = prothrombin time.

### Survival analysis

**Figure 2:**
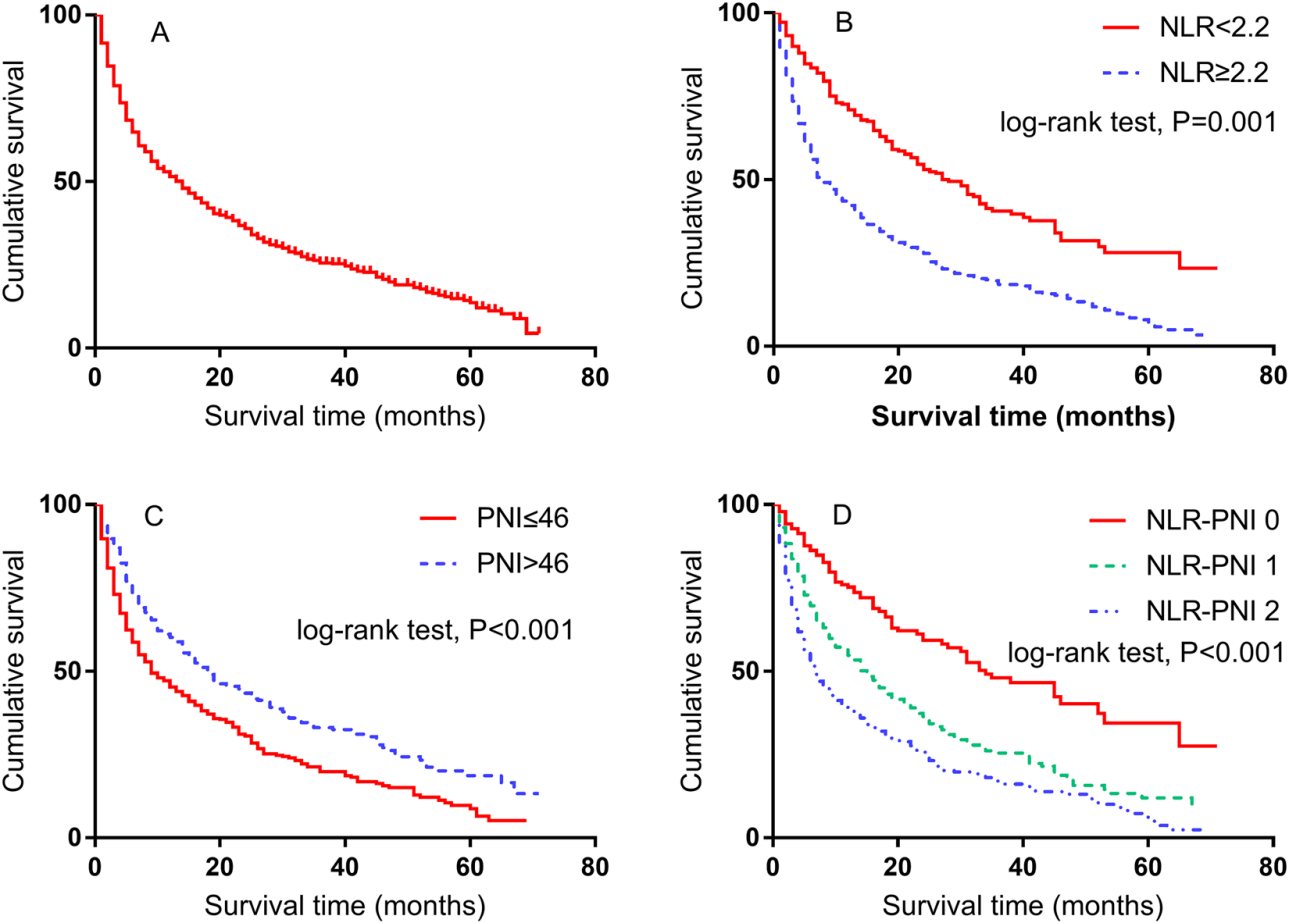
Kaplan-Meier survival curves for overall survival in 793 patients undergoing transarterial chemoembolization for hepatocellular carcinoma. (**A**) Overall survive; (**B**) NLR; (**C**) PNI; (**D**) NLR-PNI score.

The median survival duration was 15 months (range, 1–71 months). At the time of the final analysis, 587 of 793 (74.9%) patients had died of liver disease. The 1-, 3-, and 5-year OS rates were 49.9%, 25.0%, and 12.7%, respectively (Fig. 2A). The survival analysis confirmed that the inflammation-based indices and scores were strong predictors of survival, as the median OS durations for patients with a NLR ≤ 2.2 was 18 months (95% confidence interval [CI]: 22.7–33.3 months) vs. 7 months (95% CI: 5.6–8.4 months) for those with a NLR > 2.2 (Fig. 2B). Similarly, HCC patients with a PNI ≤ 46 had a median OS of 9 months (95% CI: 6.7–11.2 months) vs. 18 months (95% CI: 14.6–21.9 months) for those with a PNI > 46 (Fig. 2C). Patients in the NLR-PNI 0 group had the most favourable outcomes, with a median OS of 33 months (95% CI: 22.8–43.2 months) vs. 14 months (95% CI: 10.9–17.1 months) and 6 months (95% CI: 9.9–14.1 months) for those in the NLR-PNI 1 and NLR-PNI 2 groups, respectively.

### Univariate and multivariate analysis

The univariate OS analysis revealed significant associations of an unfavourable OS with a higher values of the following parameters: pre-treatment NLR (hazard ratio (HR), 2.20; 95% CI: 1.81–2.65; P < 0.001), TBIL (HR, 1.64; 95% CI: 1.33–2.01; P < 0.001), ALT (HR, 1.34; 95% CI: 1.14–1.58; P < 0.001), AST (HR, 1.95; 95% CI: 1.63–2.44; P < 0.001), AFP (HR, 1.57; 95% CI: 1.33–1.85; P < 0.001); Child–Pugh grade (HR, 1.33; 95% CI: 1.08–1.63; P = 0.017). OS was also found to associate with the largest tumour size (HR, 1.76; 95% CI: 1.42–2.19; P < 0.001), vascular invasion (HR, 2.05; 95% CI: 1.64–2.56; P < 0.001), and BCLC stage (HR, 2.23; 95% CI: 1.79–2.78; P < 0.001). By contrast, a higher PNI was associated with a better OS (HR, 0.525; 95% CI: 0.355–0.777; P = 0.001. However, NLR-PNI scores of 1 (HR, 1.89; 95% CI: 1.45–2.48; P < 0.001) and 2 (HR, 2.86; 95% CI: 2.20–3.72; P < 0.001) associated significantly with a worse OS (Table 3).

**Table 3:** Prognostic factors associated with overall survive.

Variables	Univariate		Multivariate	
	HR (95%CI)	P value	HR (95%CI)	P value
Age (y) (≤55, >55)	0.93(0.79–1.10)	0.399		
Gender (male/female)	1.17(0.93–1.46)	0.178		
HBsAg (positive/negative)	0.96(0.77–1.19)	0.688		
ALB, g/L (≤35, >35)	0.76(0.58–0.92)	<0.001		
TBIL, μmol/L (≤28, >28)	1.64(1.33–2.01)	<0.001	1.53(1.11–2.09)	0.009
ALT, IU/L (≤40 >40)	1.34(1.14–1.58)	<0.001		
AST, IU/L (≤35 >35)	1.95(1.63–2.44)	<0.001		
PT, sec (≤12/>12)	1.13(0.95–1.34)	0.157		
AFP, ng/mL (≤400/>400)	1.57(1.33–1.85)	<0.001		
Child-pugh grade (A/B)	1.33(1.08–1.63)	0.007		
MELD score	1.00(0.99–1.03)	0.238		
Largest tumor size (cm) (≤10/>10)	1.76(1.42–2.19)	<0.001		
Tumor number (single/multiple)	1.01(0.84–1.22)	0.888		
Vascular invasion (present/absent)	2.05(1.64–2.56)	<0.001	2.16(1.43–3.22)	0.001
BCLC (B/C stage)	2.23(1.79–2.78	<0.001	1.75(1.34–2.29)	<0.001
NLR (≤2.2, >2.2)	2.20(1.81–2.65)	<0.001		
PNI (≤46, >46)	0.66(0.56–0.78)	<0.001		
NLR-PNI				
0				
1	1.89(1.45–2.48)	<0.001	2.08(1.33–3.25)	0.001
2	2.86(2.20–3.71)	<0.001	3.92(2.11–7.26)	<0.001

According to the multivariate Cox proportional hazards model, the pre-treatment TBIL level (HR, 1.53; 95% CI: 1.11–2.09; P = 0.009), vascular invasion (HR, 2.16; 95% CI: 1.43–3.22; P = 0.001), BCLC stage (HR, 1.75; 95% CI: 1.34–2.29; P < 0.001), and a pre-treatment NLR-PNI score of 1 (HR, 2.08; 95% CI: 1.33–3.25; P < 0.001) or 2 (HR, 3.92; 95% CI: 2.11–7.26; P < 0.001) were independently predictive of OS (Table 3).

### Discriminatory performances of the staging systems and inflammation scores

**Figure 3:**
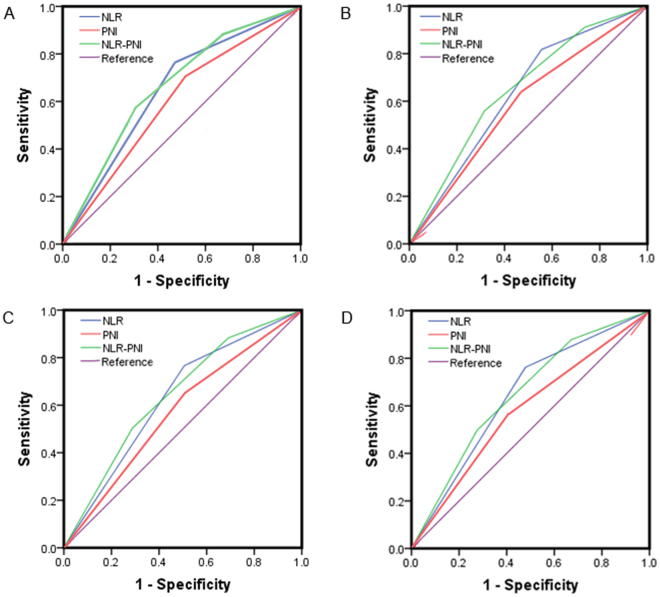
Comparisons of the area under the curve for outcome prediction. Comparisons among the inflammation-based index and scores at (**A**) Overall survive time; (**B**) at 1-year; (**C**) at 3-year; and (**D**) at 5-year in patients with hepatocellular carcinoma after TACE.

The prognostic power of each inflammation-based index and score was compared by analysing the areas under the ROC curves calculated for the patients’ survival statuses at the overall survive, 1-year, 3-year, and 5-year follow-ups (Fig. 3). As shown in Table 4, the NLR-PNI score had a superior discriminative capacity when compared with the NLR and PNI alone, as well as a consistently higher area under the curve value relative to the other inflammation-based indices.

**Table 4:** Comparison of the AUC values between the inflammation-based index and score.

Period	AUC (95%CI)	P value
Overall survive		
NLR	0.646(0.601–0.691)	<0.001
PNI	0.595(0.549–0.642)	<0.001
NLR-PNI	0.683(0.639–0.728)	<0.001
1-year		
NLR	0.631(0.592–0.670)	<0.001
PNI	0.578(0.538–0.618)	0.002
NLR-PNI	0.679(0.631–0.707)	<0.001
3-year		
NLR	0.630(0.587–0.674)	<0.001
PNI	0.574(0.530–0.617)	0.001
NLR-PNI	0.673(0.630–0.715)	<0.001
5-year		
NLR	0.642(0.597–0.687)	<0.001
PNI	0.572(0.527–0.618)	<0.001
NLR-PNI	0.679(0.635–0.723)	<0.001

## Discussion

TACE is a standard treatment for patients with unresectable BCLC-B stage HCC^3^. However, in a real clinical setting, many BCLC-C HCC patients with preserved hepatic function also undergo TACE to prolong survival and relieve clinical symptoms. Patients with unresectable disease comprise a heterogeneous population with both BCLC-B and C stage disease and a wide range of tumour burdens, and thus exhibit variable outcomes. Therefore, we believe that novel and accurate prognostic biomarkers are needed for patients undergoing TACE for unresectable HCC.

HCC has an inflammatory pathogenesis. Particularly in China, most HCC cases develop consequent to an underlying chronic HBV infection. Both tumour inflammation and immunologic factors are known to enable cancer characteristics, and increasing evidence supports the involvement of both types of factors in cancer progression and metastasis^14,15^. Many previous investigations have demonstrated a significant association of a low preoperative NLR score with a favourable OS rate in patients undergoing TACE for unresectable HCC^16,17^. Although the exact mechanisms by which a high NLR score is associated with a poor outcome remain unknown, the following provides a potential explanation: first, cytokines involved in cancer-associated inflammation, such as interleukin-6 (IL-6) and tumour necrosis factor-α (TNFα), may induce neutrophilia^18^. These induced neutrophils, together with circulating neutrophils, may secrete proangiogenic factors that leading to angiogenesis and tumour progression and thus predict a poor clinical outcome. Second, lymphocytes are important components of the adaptive immune system that provide a cellular basis for cancer immuno-surveillance and immuno-editing, and evidence has proven that infiltrating lymphocytes suggest the generation of an effective anti-tumour cellular immune response. In other words, a lower lymphocyte count might indicate an inadequate immunological anti-tumour response and a weakened defence against cancer, with a consequently poor prognosis^19^.

The PNI, which is calculated from the serum ALB level and total peripheral blood lymphocyte count, was originally proposed to assess the perioperative immunonutritional status and surgical risk in patients undergoing gastrointestinal surgery. However, this index has recently been used to assess the immunonutritional conditions of patients with different types of cancer. HCC and other cancers of the liver, an important metabolic organ, have been associated with an increase in malnutrition, as well as concomitant underlying cirrhosis that might weaken anti-tumour and anti-metastasis responses. A low PNI value indicates a relatively poor nutritional status and lymphocytopaenia, and studies have shown that PNI is closely related to tumorigenesis and cancer progression in a variety of malignancies^20–22^. For example, Chan *et al*.^23^ analysed patients with BCLC 0/A HCC who underwent surgical resection and found that the PNI was a significant prognostic factor for OS in patients undergoing curative surgery for very early-/early-stage HCC. Furthermore, Okamura *et al*.^24^ compared the power of various prognostic scores, including the Glasgow Prognostic Score (GPS), NLR, and PNI, and demonstrated that NLR and PNI were predictors of OS in patients undergoing hepatectomy with curative intent for HCC.

As the prognostic values of the NLR and PNI are stable in patients with early-stage HCC, we evaluated whether a combination of these scores could optimize the selection of patients who would benefit from TACE among a patient subgroup with varying tumour burdens and variable predicted survival outcomes. Our results indicated that the combined NLR-PNI score could better reflect the systemic inflammatory response for patients with HCC after TACE, compared with either score alone. In our study, the performances of NLR and PNI alone as prognosticators were overtaken by the performance of the integrated NLR-PNI score. After dividing our patients into three groups according to NLR-PNI scores, we found that those with high combined scores had progressively worse outcomes relative to those with lower scores, and these differences were largely significant.

Previous studies hypothesized that the PNI reflects the patient’s general status, including their immunonutritional status, liver function, and immune activity, rather than the malignant potential of the tumour^25^, and this was supported by the lack of significant differences between the low and high preoperative PNI groups with respect to various tumour factors, including the preoperative AFP and DCP levels and tumour diameter. To our knowledge, our study is the first to evaluate the ability of the combined NLR-PNI score to predict the outcomes of patients with intermediate-to-advanced HCC. We note that elevated scores are more frequently observed in patients with vascular invasion, a larger tumour size, and an advanced clinical stage, suggesting that a systemic inflammatory response can predict a more aggressive clinical phenotype. Simultaneously, higher TBIL and AST levels, a prolonged PT, and a high Child–Pugh grade were associated with worse hepatic function, suggesting that a systemic inflammatory response is predictive of a more aggressive clinical phenotype and liver dysfunction.

This study had some potential limitations of note. First, this was a retrospective, single-centre study. Second, only TACE-treated patients were enrolled. Third, many other biologic markers, including the C-reactive protein level, GPS, and platelet-to-lymphocyte ratio (PLR), were not included and compared, although they have been proposed as prognostic factors for patients with HCC. Further studies are needed to determine which marker(s) could better reflect a poor systemic inflammatory state or whether a combination of markers could improve the prognostic ability.

## Conclusion

In conclusion, the present study revealed that a higher NLR-PNI score predicted a worse prognosis in patients who underwent TACE for unresectable HCC. Our results suggest that the NLR-PNI score could serve as a novel, easily available, and inexpensive inflammation-based index in routine clinical practice.

## References

[1] Jemal A (2011). Global cancer statistics. CA Cancer J Clin..

[2] Peng W (2015). Prognostic value of the platelet to lymphocyte ratio change in liver cancer. J Surg Res..

[3] EASL-EORTC clinical practice guidelines: management of hepatocellular carcinoma. *J Hepatol*. **56**, 908–943 (2012). 10.1016/j.jhep.2011.12.001 22424438

[4] Llovet JM (2002). Arterial embolisation or chemoembolisation versus symptomatic treatment in patients with unresectable hepatocellular carcinoma: a randomised controlled trial. Lancet..

[5] Llovet JM, Bruix J (2003). Systematic review of randomized trials for unresectable hepatocellular carcinoma: Chemoembolization improves survival. Hepatology..

[6] Pinato DJ, North BV, Sharma R (2012). A novel, externally validated inflammation-based prognostic algorithm in hepatocellular carcinoma: the prognostic nutritional index (PNI). Br J Cancer..

[7] Huang ZL, Luo J, Chen MS, Li JQ, Shi M (2011). Blood neutrophil-to-lymphocyte ratio predicts survival in patients with unresectable hepatocellular carcinoma undergoing transarterial chemoembolization. J Vasc Interv Radiol..

[8] Kinoshita A (2012). Comparison of the prognostic value of inflammation-based prognostic scores in patients with hepatocellular carcinoma. Br J Cancer..

[9] He CB, Lin XJ (2017). Inflammation scores predict the survival of patients with hepatocellular carcinoma who were treated with transarterial chemoembolization and recombinant human type-5 adenovirus H101. PLOS ONE..

[10] Bruix J, Sherman M (2005). Management of hepatocellular carcinoma. Hepatology..

[11] Llovet JM, Bru C, Bruix J (1999). Prognosis of hepatocellular carcinoma: the BCLC staging classification. Semin Liver Dis..

[12] Xiao GQ, Liu C, Liu DL, Yang JY, Yan LN (2013). Neutrophil-lymphocyte ratio predicts the prognosis of patients with hepatocellular carcinoma after liver transplantation. World J Gastroenterol..

[13] Onodera T, Goseki N, Kosaki G (1984). [Prognostic nutritional index in gastrointestinal surgery of malnourished cancer patients]. Nihon Geka Gakkai Zasshi..

[14] Colotta F, Allavena P, Sica A, Garlanda C, Mantovani A (2009). Cancer-related inflammation, the seventh hallmark of cancer: links to genetic instability. Carcinogenesis..

[15] Hanahan D, Weinberg RA (2011). Hallmarks of cancer: the next generation. Cell..

[16] Zhou D (2016). Derived neutrophil to lymphocyte ratio predicts prognosis for patients with HBV-associated hepatocellular carcinoma following transarterial chemoembolization. Oncol Lett..

[17] Qi X (2016). Neutrophil-to-lymphocyte ratio for the prognostic assessment of hepatocellular carcinoma: A systematic review and meta-analysis of observational studies. Oncotarget..

[18] Ulich TR, Del CJ, Guo KZ (1989). *In vivo* hematologic effects of recombinant interleukin-6 on hematopoiesis and circulating numbers of RBCs and WBCs. Blood..

[19] Hoffmann TK (2002). Spontaneous apoptosis of circulating T lymphocytes in patients with head and neck cancer and its clinical importance. Clin Cancer Res..

[20] Hong S (2015). The prognostic nutritional index (PNI) predicts overall survival of small-cell lung cancer patients. Tumour Biol..

[21] Jiang N (2014). The role of preoperative neutrophil-lymphocyte and platelet-lymphocyte ratio in patients after radical resection for gastric cancer. Biomarkers..

[22] Mohri Y (2013). Prognostic nutritional index predicts postoperative outcome in colorectal cancer. World J Surg..

[23] Chan AW (2015). Prognostic Nutritional Index (PNI) Predicts Tumor Recurrence of Very Early/Early Stage Hepatocellular Carcinoma After Surgical Resection. Ann Surg Oncol..

[24] Okamura Y (2015). Preoperative neutrophil to lymphocyte ratio and prognostic nutritional index predict overall survival after hepatectomy for hepatocellular carcinoma. World J Surg..

[25] Okamura, Y. *et al*. The optimal cut-off value of the preoperative prognostic nutritional index for the survival differs according to the TNM stage in hepatocellular carcinoma. *Surg Today* (2017). 10.1007/s00595-017-1491-0 28315008

